# Dynamic Operations in Macromolecular Data Storage

**DOI:** 10.1002/anie.5128253

**Published:** 2026-08-24

**Authors:** Jakub Ossowski, Oskar Affenberger, Jean‐François Lutz, Maria Nerantzaki

**Affiliations:** ^1^ CNRS ISIS Université de Strasbourg Strasbourg France

**Keywords:** digital polymers, DNA data storage, DNA nanotechnology, informational polymers, sequence‐controlled polymers

## Abstract

In recent years, numerous concepts have been reported for storing data on molecules, either small molecule mixtures, oligomer libraries or polymers. In the latter case, binary information is stored in a controlled monomer sequence. This can be achieved in biological polymers such as DNA but also in a variety of synthetic macromolecules. However, most existing approaches focus on passive “cold” storage, in which macromolecules serve as static archives. This minireview explores recent advances in reconfigurable macromolecular storage, highlighting strategies for erasing, rewriting, computing, repairing, or sorting‐out binary‐encoded macromolecules. All these options are discussed herein for both natural and synthetic polymers.

## Graphical abstract

With the exponential growth of digital data and the limitations of conventional silicon‐based storage and computing technologies, macromolecular data storage, the practice of encoding digital information within large, complex molecules like DNA or synthetic polymers, has emerged as a scalable and sustainable alternative to traditional digital technologies. This minireview provides an overview of recent approaches to enable dynamic control in molecular data storage. While challenges remain, ongoing advancements in editing, erasing, and rewriting, bring this transformative technology closer to practical, large‐scale use, bridging the gap between chemistry, biology, computer science, and engineering techniques.

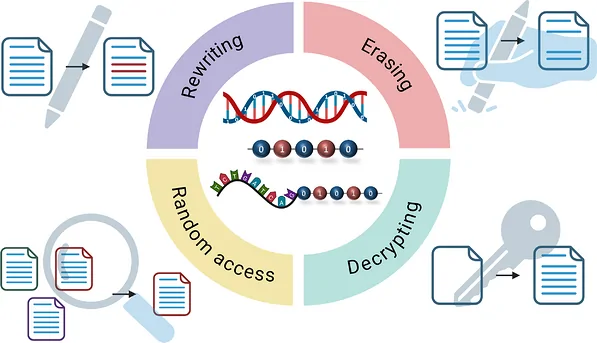

## 1 Introduction

With the increasing volume of data that are produced in our modern world, the need for alternative storage media has become a prominent topic [1]. Among the proposed alternatives, molecular storage has emerged as a promising option. In these strategies, data is stored using small molecules mixtures [2, 3, 4], oligomers [5, 6], or polymers [7, 8]. In the case of macromolecular storage, the sequence of monomers within a polymer chain serves as the medium for information encoding (Figure 1) [9]. This approach is particularly powerful because nature has already validated it: DNA, the biological “hard drive” of life, stores genetic information using a four‐letter alphabet. Inspired by this natural model, researchers have explored DNA as a technological solution for data storage (Figure 1a) [10, 11]. In parallel, other macromolecules have been investigated for molecular storage [12], including biopolymers like proteins [13, 14], as well as a wide range of synthetic polymers developed over the past decade (Figure 1a) [15, 16]. These information‐containing macromolecules are collectively referred to as digital polymers.

**FIGURE 1:**
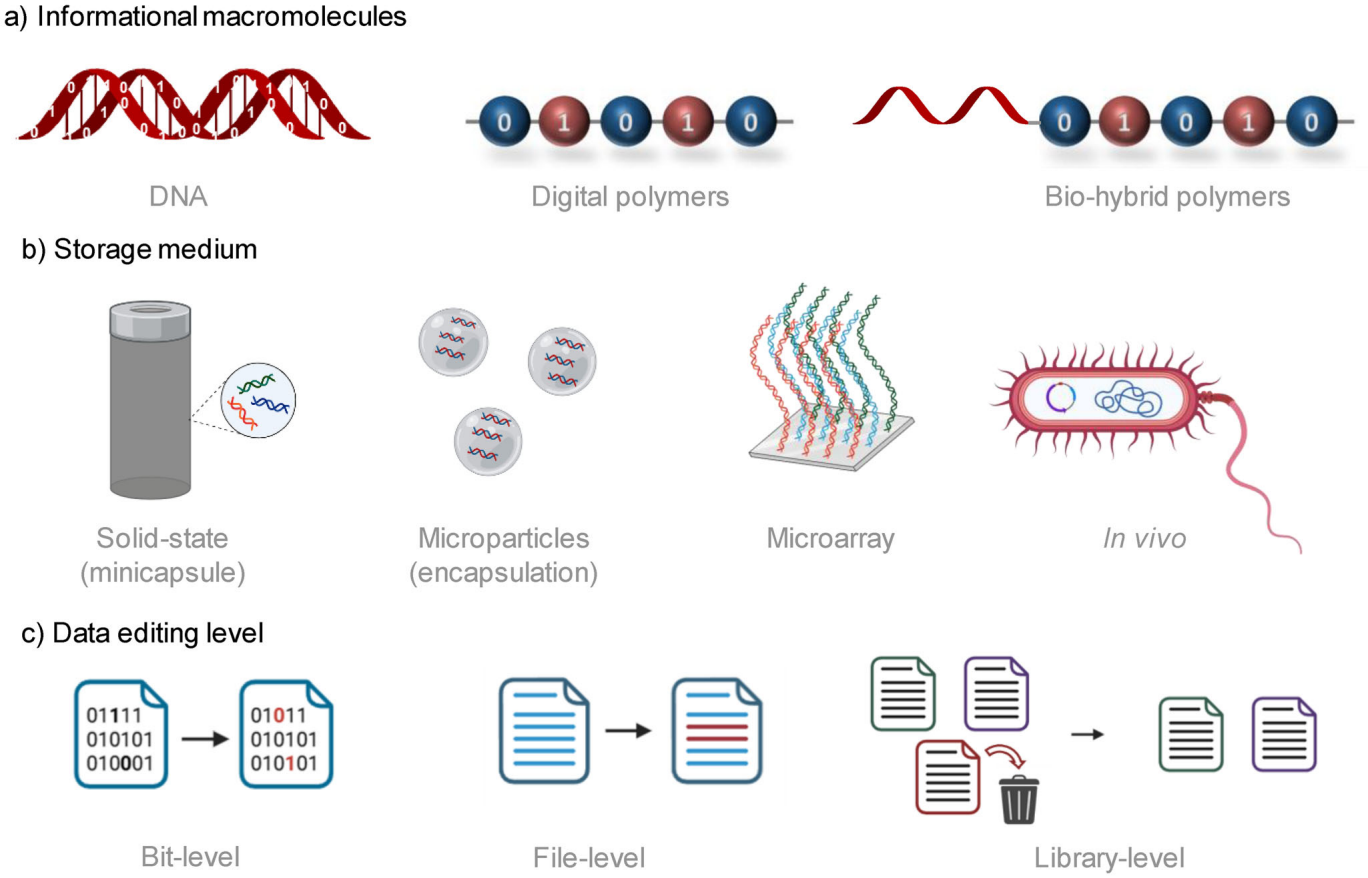
General aspects of macromolecular data storage. (a) Different types of macromolecules allowing information storage. (b) Examples of storage media for digital macromolecules. (c) Different types of data editing that can be achieved in macromolecular data storage.

Macromolecular data storage generally relies on three core features: writing, storing, and reading. Writing is typically achieved through chemical or enzymatic polymer synthesis [15], and reading is performed via sequencing [17]. These foundational principles were first demonstrated at the single‐chain level for both DNA [18] and synthetic polymers [19, 20]. At a higher level of organization, the spatial arrangement of polymer libraries has been explored to achieve greater storage capacities (Figure 1b). For digitally encoded DNA, this includes the use of microarrays [21], pooled DNA libraries [22], encapsulated libraries [23, 24, 25], DNA origami [26], and bacterial storage [27]. In the case of synthetic polymers [28], mixtures [29, 30], well plates [31], microarrays [32], suprastructures [33], crystals [34], and multilayered materials [35] have been investigated. In general, these new storage media are primarily designed for cold storage, such as archival purposes, and long‐term data storage [23].

However, macromolecular data storage is not limited to passive functions and can also include dynamic data operations (Table 1) [7, 12]. Through stimuli‐responsive chemistry [36], dynamic chemistry [37], macromolecular editing [38], and strand‐exchange mechanisms [39], advanced storage features like random access, erasure, rewriting, or error correction become possible. In recent years, multiple strategies for editing macromolecular information in DNA or synthetic polymers have been reported. As illustrated in Figure 1c, these strategies operate at different editing levels. At the most fundamental level (bit level), the informational monomer sequence can undergo chemical modification. Additionally, individual polymer chains (file level) or multiple chains (library level) can be exchanged. This minireview aims to outline, categorize and critically evaluate these novel options.

**TABLE 1:** Non‐exhaustive list of operations (beyond read/write) addressed in this minireview.

Operation	Definition	Reversibility
Erasure	Permanently deleting data	Irreversible
Rewriting	Overwriting data to replace content	Irreversible^a^
Copying	Duplicating data to another location	Reversible
Repair	Fixing errors in datasets	Irreversible^b^
Random access	Directly accessing any data point	Reversible
Sorting	Arranging data in a specific order	Reversible
Computation	Process of calculating or solving	Reversible

^a^ Unless backed up.

^b^ Unless changes are tracked.

## 2 Dynamic Operations in DNA‐Based Data Storage

To execute dynamic operations on DNA‐based data storage, multiple strategies can be employed. These include epigenetically inspired single‐bit modifications and enzyme‐mediated operations, as well as toehold‐mediated strand displacement (TMSD)‐based mechanisms [39]. Additionally, multiple triggers (such as primers, light, temperature, chemical signals, or electric fields), can be used to enable rewritable, addressable, and scalable DNA storage. Biological mechanisms like CRISPR‐Cas9 gene editing and recombinant DNA allow for adaptive data manipulation, effectively transforming cells into living data storage and processing units. All these options are briefly summarized below.

### 2.1 Epigenetic‐Inspired Editing

As shown in Figure 1c, the most basic strategy for editing macromolecular information is the direct modification of the monomer sequence. In the case of DNA, this can be achieved through epigenetic‐like modifications, which play a vital role in regulating information in biological systems. Likewise, DNA storage‐media can utilize modified nucleotides to encode digital information [40]. Beyond static encoding, controlled chemical transformations of nucleobases are able to dynamically alter stored data. Balasubramanian and coworkers demonstrated that multiple binary information layers can be stored within a single DNA template by combining canonical DNA bases with chemically modified derivatives of cytosine (Figure 2a) [41]. To retrieve the hidden layers of information, the DNA is subjected to chemical treatments. The decoding relies on bisulfite‐mediated hydrolytic deamination, which converts cytosine to uracil. In contrast, 5‐methylcytosine is resistant to deamination and therefore remains unaffected. Uracil is subsequently read as thymine after polymerase chain reaction (PCR) amplification, thereby switching the bit state. This strategy can be extended to a three‐layer system by incorporating 5‐hydroxymethylcytosine, which shows different sensitivity to deamination after prior oxidation with potassium perruthenate. Accordingly, Schroeder, Milenkovic and coworkers presented a two‐dimensional DNA data storage medium that stores main data in DNA sequence (A/C/G/T) and metadata in DNA backbone “nicks” (physical cuts) [42]. In this case, nicking was also used to edit metadata and control information (like IDs, states, and indexing signals) rather than the main image content, allowing minimal updates without resynthesizing DNA.

**FIGURE 2:**
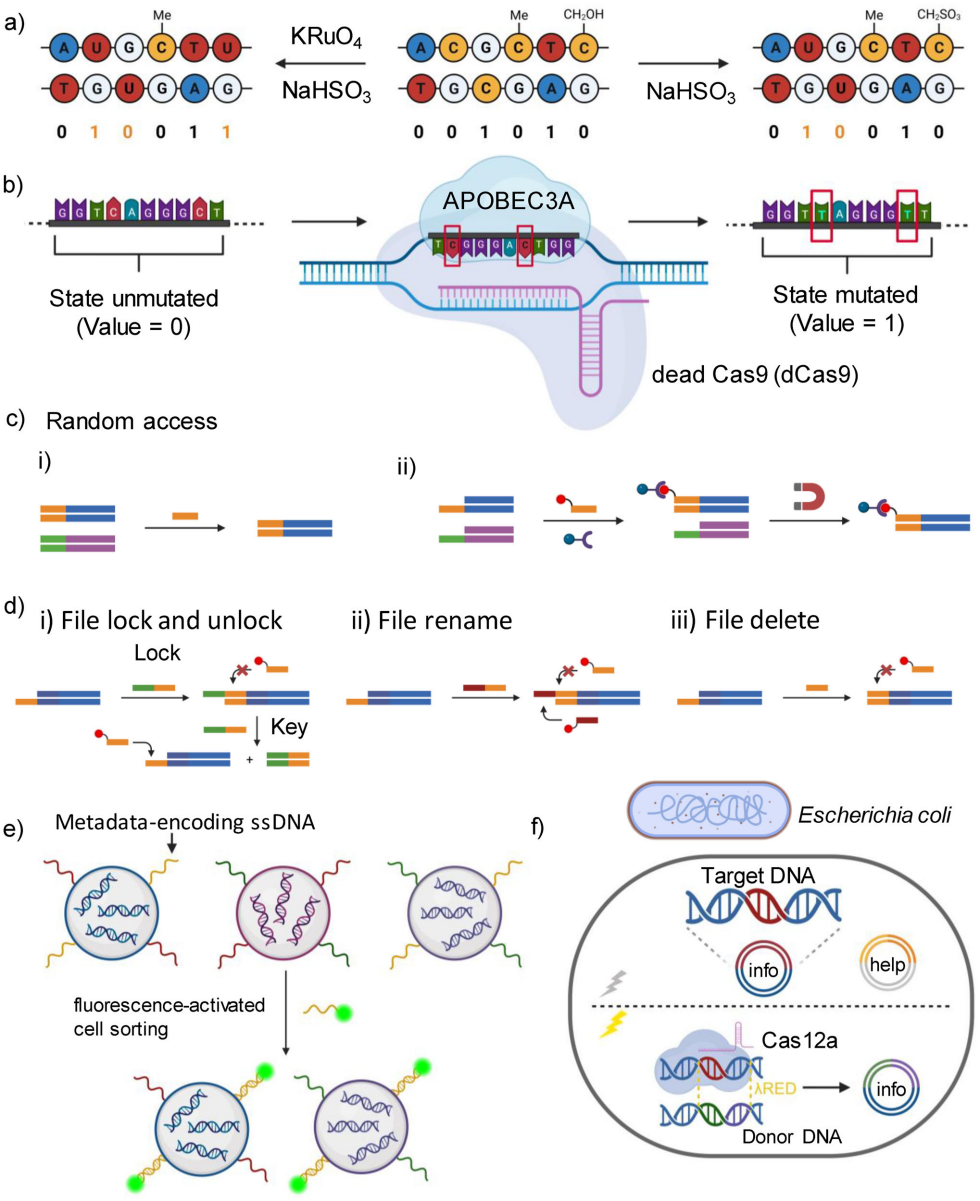
Overview of strategies enabling dynamic operations in DNA‐based data storage. (a) Epigenetic‐like modifications through chemical treatment [41]. (b) Digital data storage on DNA tape using in vitro CRISPR base‐editing reactions [43]. (c) Random access via (i) primer‐addressing and (ii) physical separation (d) ssDNA overhangs allowing in‐storage file operations including (i) lock, unlock, (ii) rename, and (iii) delete through hybridization and strand displacement reactions [44]. (e) Encapsulation‐based DNA file system for scalable indexing, retrieval, and Boolean selection [45]. (f) A dual‐plasmid system based on gene editing tools introduced into *Escherichia coli* for accurate information processing [46].

### 2.2 Enzyme‐Mediated Operations

Alternatively, to epigenetic‐like editing, Nivala and coworkers proposed very recently a CRISPR‐Cas9 strategy as an efficient enzymatic tool enabling programmable, sequence‐specific targeting [47]. In this approach, each file in the DNA pool is assigned a unique primer binding site (PBS) and data retrieval relies on PCR amplification using primers complementary to a unique PBS. Guide RNAs (gRNAs) direct Cas9 to cut or enrich only strands containing matching address sequences. This CRISPR‐Cas9‐based approach does not synthesize new DNA during retrieval but instead, uses programmable cutting to selectively access and semantically search pre‐stored DNA data, turning DNA storage into a searchable molecular database. CRISPR base‐editing tools were also used to develop a DNA Mutational Overwriting Storage system (DMOS) that stores and edits data by chemically mutating DNA at specific positions (Figure 2b). This medium stores data by treating DNA like a memory chip, where CRISPR base editors act as addressable, independent “write operations” that mutate specific bases on a pre‐synthesized DNA tape to encode digital information [43]. A different mechanism that enables selective deletion of stored metadata using the CRISPR‐Cas12a was reported by Song and coworkers [48]. In this study, CRISPR‐Cas12a's collateral cleavage activity is used to selectively and irreversibly degrade DNA‐based metadata, enabling secure sanitization of stored files while leaving the primary data intact.

In the same context, Endy and coworkers introduced a mechanism for site‐specific editing of DNA‐encoded information that relies on enzyme‐mediated recombination [27]. The concept uses site‐specific recombinases (e.g., Cre or Flp enzymes) to cut and paste DNA segments at specific recognition sites (e.g., loxP or FRT sites). This allows for rewriting information at arbitrary locations within the DNA storage blocks. Velev, Keung, and coworkers also described a system that enables selective erasure and rewriting of stored DNA information through addressable molecular control [49]. In this case, each DNA “file” is designed with a unique sequence‐based address that allows it to be specifically targeted within a larger data pool. These addresses act like molecular barcodes, enabling complementary probes or programmable promoter mechanisms to recognize and activate only the intended region of data. Once a target file is identified, the existing information can be selectively removed. In brief, restriction endonucleases first introduce precise cuts at engineered recognition sites within the target DNA, while exonucleases subsequently digest the resulting fragments by progressively degrading the exposed DNA strands.

Very recently, Liu and coworkers proposed a modular DNA storage approach, which enables programmable assembly, logical rewriting, and hierarchical access of digital information stored in DNA [50]. In this system, the information is not synthesized in one block but it is assembled using premade DNA fragments. These fragments, referred to as DynaBytes, are synthesized with defined terminal linkers and 5'‐phosphorylation, enabling their directional ligation through the action of T4 DNA ligase. In fact, these terminal modifications allow the assembly into complete storage units (e.g., 1–10 kb), by sequence‐specific terminal complementarity. Moreover, due to their well‐engineered termini (e.g., sticky ends for ligation or restriction enzyme recognition sites), DynaBytes can be selectively joined or cleaved, enabling the assembly of larger data structures as well as the restructuring and updating of existing datasets.

### 2.3 Strand Displacements

Beyond the nucleotide modifications and the enzyme‐mediated editing in DNA data storage described in Sections 2.1 and 2.2, exchanging complete informational strands represents an appealing strategy for macromolecular editing. In this context, TMSD is a powerful tool for rearranging DNA strands with high precision and addressability [51]. TMSD is a molecular process where a short single‐stranded DNA or “toehold” binds to a complementary region of a double‐stranded nucleic acid, initiating the displacement of an existing strand. For instance, TMSD is widely used in the field of DNA computing [52]. Nevertheless, strategies that combine DNA data storage with computation abilities remain scarce [53, 54]. One example is single instruction multiple data DNA computation, which enables molecular operations to be performed directly within the storage medium [55]. In this approach, 4‐bit data registers consist of a bottom strand hybridized to several top strands, with information encoded in the spatial pattern of nicks. Operations are carried out by introducing a set of instruction strands that initiate hybridization and strand displacement reactions across the entire pool of registers. After each operation, unreacted strands and waste products are removed via magnetic bead separation. The system can execute programs such as binary counting and the Turing universal cellular automaton Rule 110. The platform supports multiple consecutive rounds of computation, as well as random access for the selective retrieval and permanent erasure of specific data files.

TMSD has also been widely used to manipulate information in various DNA‐based storage. However, in most of these approaches, DNA serves as a nanoscale organizational tool rather than a medium for encoding digital information directly in its nucleotide sequence. In other words, binary states (0/1) are not represented by nucleotides themselves, but by other entities, such as the distinction between single‐ and double‐stranded DNA molecules [56], sequence‐specific hybridization rules [57], bulky DNA junctions [58], DNA nanoswitches [59], fluorescent signals [60, 61, 62], or biotin‐avidin complexes [63, 64]. As these concepts operate at the nanoscale rather than at the molecular scale, they fall outside the scope of this review. Nevertheless, readers are directed to representative examples for further reference [26, 65, 66].

### 2.4 Random and Hierarchical Access

PCR is the primary technique used to enable random access in DNA data storage (Figure 2c). This is achieved by flanking each DNA data sequence with specific primer‐binding regions, allowing selective amplification and subsequent sequencing of target files from complex DNA libraries [44, 67, 68, 69, 70, 71, 72]. For instance, it has been shown that 35 distinct files can be individually retrieved without errors from a library containing more than 13 million DNA oligonucleotides using this approach [73]. PCR‐based random access is also scalable, reliable, and physically feasible even in large DNA databases containing more than 10^10^ unique sequences per microliter. Nevertheless, its reliability is ultimately limited by the number of physical DNA molecules sampled from the pool, rather than by the specificity of the address system used to distinguish files. As PCR amplification depletes the original DNA pool, limiting repeated access to the same data, several physical separation strategies have been proposed to isolate target files while preserving the rest of the library (Figure 2c). In that regard, Keung and coworkers suggested using biotin‐labelled primers for PCR‐based selection, followed by magnetic bead‐based removal of the amplified targets [73]. This approach was further improved by introducing a T7 promoter domain and a single‐stranded DNA (ssDNA) overhang to the data strands [44]. The T7 promoter is a short, specific DNA sequence that is recognized by the T7 RNA polymerase, leading to non‐destructive in vitro transcription. Additionally, dynamic file operations, including locking, unlocking, renaming, and deletion, can be implemented through hybridization or strand displacement at ssDNA overhangs (Figure 2d).

Nested polymerase chain reaction (nested PCR) is a two‐step amplification technique used to enhance both the specificity and sensitivity of DNA detection. It involves two successive rounds of PCR, where the second set of primers binds within the first PCR product, enabling more precise amplification and selective recovery of target sequences from complex mixtures [74]. Conclusively, while PCR‐based random access enables direct retrieval of individual DNA‐encoded files via unique sequence addresses, hierarchical access using nested PCR offers a scalable, multi‐level framework for structured data retrieval, though it introduces greater complexity.

Moreover, data access is achieved not only through sequence‐specific addressing but also by tuning the thermodynamic properties of DNA hybridization. Unlike conventional PCR‐based approaches that rely on strictly orthogonal primer addresses, this strategy introduces a more flexible and scalable framework for organizing and accessing DNA‐encoded information. By modulating environmental parameters such as temperature and strand concentration, selective subsets of DNA sequences can be retrieved from complex libraries [75]. This enables hierarchical and partial data access, such as the retrieval of low‐resolution “file previews” prior to full data extraction. Zhang and coworkers used thermal stimuli to enable rapid and permanent data erasure in DNA libraries [76]. In this approach, true messages are hybridized with truth markers, while false messages are bound to chemically modified false markers that prevent PCR amplification. Thermal denaturation followed by stochastic rehybridization disrupts the original connections between messages and markers, making the data unrecoverable. Overall, these thermal methods act as global triggers that lack the selectivity required for complex data operations.

### 2.5 Scalable and Structured DNA Data Management

In an early study, Strauss, Ceze, and coworkers proposed a pioneering concept for scalable and structured DNA data management in which data are stored as free‐floating DNA strands. A machine‐learned encoding strategy is used to map information into DNA sequences such that molecular hybridization strength directly enables similarity‐based retrieval of data [77]. Bathe and coworkers introduced a DNA data storage system that extends similarity‐driven data retrieval by encoding DNA files as physically “tagged and sortable” particles (Figure 2e) [45]. In this approach, DNA sequences are encapsulated within silica capsules and labelled on the surface with ssDNA barcodes. File identification is achieved through hybridization of fluorescently labelled probes to these barcodes, enabling high‐throughput retrieval via fluorescence‐activated cell sorting. The system further supports Boolean logic queries, allowing subsets of files to be selected based on combinations of metadata barcodes. Instead of retrieving DNA data using hybridization, Zhang and coworkers described a system that supports logic‐controlled molecular selection of data [78]. In this approach, molecular logic circuits (DNAzymes) control gold nanoparticle‐based “folders” to selectively retrieve information on demand. Fluorescently labelled, calcium phosphate‐coated silica particles that encapsulate DNA data and function as physically addressable barcoded storage units for multiplexed random access have been also proposed by Lu and coworkers [79]. In this case though, “editing” is performed by selectively retrieving DNA from particles, modifying it externally, and then re‐encapsulating the updated DNA rather than directly rewriting sequences inside the microspheres.

Jiang and coworkers demonstrated that binary encoded ssDNA strands can be either spooled or linearly arranged on a substrate like a polymer film, mimicking cassette tape reels [80]. The resulting DNA “tape” is divided into addressable segments, enabling targeted reading, erasing, or rewriting of specific data chunks. Cleavable linkers (*e.g*., photocleavable or enzyme‐sensitive bonds) allow for reversible attachment, making the system rewritable, sustainable, and dynamically manageable. Interestingly, data management is fully automated using a microfluidic tape‐drive system [80]. Instead, Song and coworkers reported the use of photonic microspheres with unique optical signatures, allowing DNA‐encoded data to be accessed through database‐like queries [81]. Each photonic microsphere serves as an addressable unit, assigned a unique optical index—such as a specific wavelength of light it reflects or emits. Just as files in a digital system are organized by names or metadata, each microsphere “container” holds a specific DNA sequence, allowing for hierarchical organization of data. Lately, the same research group also introduced a versatile, low‐cost, and portable paper‐based DNA storage system that supports spatial addressing [82]. This means specific regions of the paper can be individually erased or rewritten without affecting the rest of the substrate, enabling precise and dynamic data management. To erase the stored data, the system employs chemical, thermal, or light‐induced methods. While it may not match the density or long‐term stability of photonic microspheres, its reprogrammability and simplicity give it a unique advantage in specific use cases. Liu and coworkers proposed an architecture in which DNA‐encoded data is immobilized in proximity to an electrode surface [83]. Electrical stimulation first gates DNA accessibility by modulating hybridization and exposure of address regions, and the resulting selected DNA template can then be enzymatically read out and amplified via T7 promoter–driven transcription into RNA signals. Electrical addressing provides structure and scalability, and T7 transcription serves as an enzymatic readout layer for accessed DNA information. In a very recent study [84], Huang, Eung, Song, and coworkers have described an electric field‐driven DNA memory chip that allows random access and permits to significantly reduce time‐consuming PCR workflows. In this new approach, spatially‐controlled DNA synthesis is obtained through electric field‐guided hybridization, which differs from classical electrode chemical synthesis.

### 2.6 In Vivo Processing

In vivo data storage is based on storing information in DNA within living cells such as bacteria and yeast, offering extremely high data density and long‐term stability. Notably, the first demonstration of rewritable biological memory in living cells was reported by Endy and coworkers in *Escherichia coli* [27]. In brief, the authors engineered a recombinase addressable data (RAD) module that allows reliable, reversible writing of digital information into the DNA of living cells. The RAD module uses phage integrases (like Bxb1) and excisionases to control the directionality of DNA recombination, enabling the flipping of a DNA sequence between two binary states. Liu and coworkers proposed an in vivo dual‐plasmid system that enables reliable storage, amplification, and rewriting of digital data encoded in DNA within living cells (Figure 2f) [46]. This system employs CRISPR‐Cas12a to cut the target plasmid in vitro, ensuring precise and targeted modification of the stored DNA. Interestingly, following this approach, the authors managed to rewrite a message using a new piece of DNA (“donor DNA”) which could be introduced via electroporation, a technique that uses brief electric pulses to open pores in cell membranes so DNA can enter. It was also shown that the rewritten information could be amplified and maintained within the cell population.

The cell disk concept, which was introduced in 2024, enables both random access and rewriting by organizing yeast cells into hard drive–like data blocks [85]. Each block is protected by a “lock‐and‐key” module based on CRISPR/Cas9. Notably, each data block can be accessed selectively by inserting a specific gRNA inducing a cleavage and reparation mechanism by homologue recombination. As such, each yeast cell acts as a miniature disk capable of storing and managing digital data at the molecular level. Unlike previous in vivo DNA storage systems, the Cell Disk supports both random reading and rewriting of data, which are essential for practical, large‐scale applications. Additionally, the encoded information can be specifically rewritten using counter‐selection by activating the URA3 gene in unlocked cells and treating them with 5‐fluoroorotic acid. After eliminating the specific cell encoding the information to be deleted, a new cell containing the updated data can be inserted into the Cell Disk.

## 3 Dynamic Operations in Polymer‐Based Data Storage

As introduced earlier, synthetic digital polymers represent an interesting alternative to DNA for macromolecular data storage. Conceptually, they share DNA's core principle: a controlled sequence of monomers. However, synthetic informational polymers remain at an earlier stage of development compared to DNA and present certain limitations. For example, unlike DNA, they cannot be amplified [86], which restricts their use at low concentrations and limits the analytical methods available for decoding. To date, only nanopore sensing, kinetically controlled chemical degradation, and tandem mass spectrometry have been successfully applied to sequence synthetic polymers [87]. While tandem mass spectrometry is currently the most efficient decoding method [88], it is both destructive and dependent on large‐scale instrumentation. On the other hand, the chemical structure of synthetic macromolecules can be far more diverse than that of nucleic acids. Indeed, an almost infinite pool of building blocks and chemical reactions can be used to construct them. Consequently, these synthetic informational macromolecules can be edited more easily at the molecular level than DNA (Figure 3). Moreover, conjugating these polymers with DNA (via bioconjugation) provides additional possibilities for tuning informational content. These recent advances are summarized in the following sections.

**FIGURE 3:**
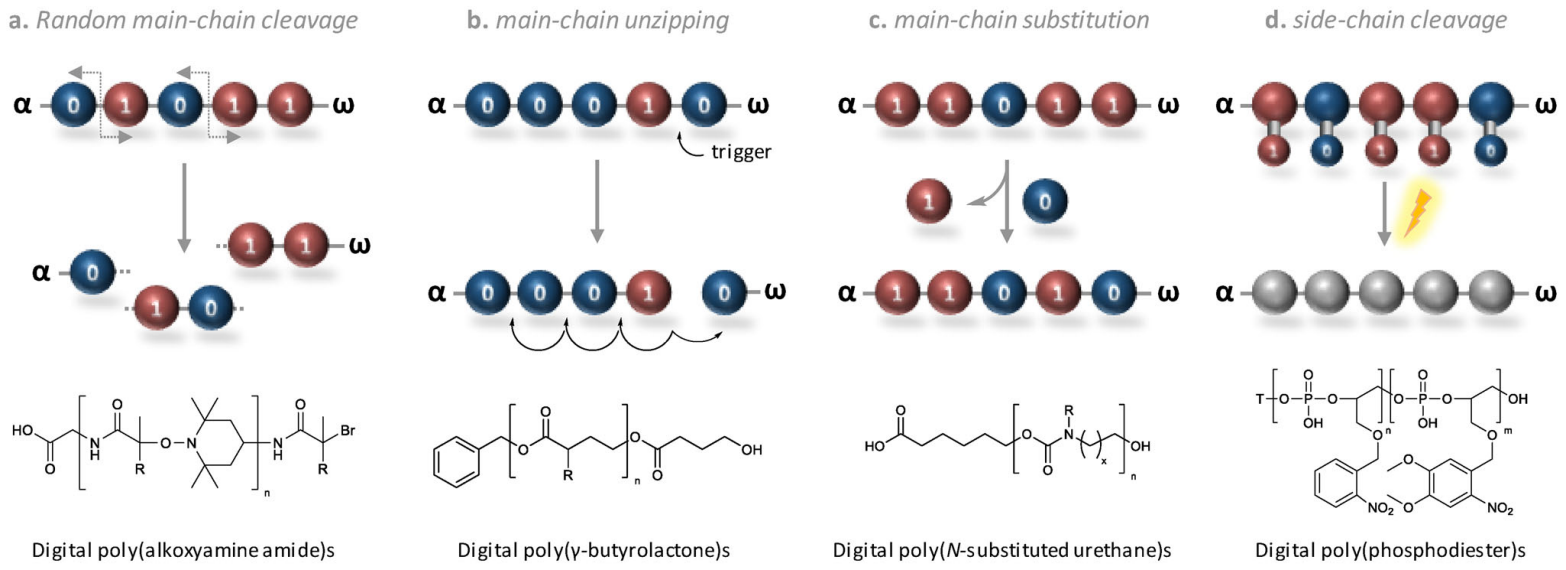
Various mechanisms that have been explored for altering the information content of synthetic digital polymers. The molecular structures shown under each category are examples of polymers that undergo these mechanisms. In panel d, the letter T represents a thymidine nucleoside end‐group.

### 3.1 Main‐Chain Random Scission

One of the simplest ways to alter synthetic digital polymers is through random chain‐scission (Figure 3a), a well‐known mechanism in polymer degradation. This process is typically non‐selective and can disrupt encoded information in sequence‐defined polymers. Overall, for polymer degradation through chain scission, the choice of trigger (e.g., light, temperature or chemical agents) determines the precision and control of the process. An early study of our group demonstrated that temperature can be used as a simple trigger to erase the digital information stored in digital poly(alkoxyamine amide)s [20]. For this purpose, sequence‐encoded oligomers were synthesized by solid‐phase synthesis and were afterwards heated at different temperatures. Size‐exclusion chromatography (SEC) measurements revealed that the encoded information can be erased by heating the polymers in the solid state or in solution, due to the homolysis of the C─ON alkoxyamine bonds. Still this sequence‐encoded polymer exhibits stability at room temperature, showing no signs of degradation even after several months of storage.

Du Prez and coworkers have developed dynamic, sequence‐defined oligomers with a chemically encoded PIN code, by combining the Passerini three‐component reaction with the thermoreversible addition of 1,2,4‐triazoline‐3,5‐diones (TADs) to indole substrates [89]. Upon heating, the TAD‐indole covalent bonds undergo random reshuffling within the polymer backbone, scrambling the encoded information and encrypting the original sequence. Decryption is achieved through a second heating step, which allows the macromolecular PIN code to be deciphered using electrospray ionization mass spectrometry (ESI‐MS). This encryption/decryption approach could be applied to macromolecular tagging of banknotes, offering a novel solution for anti‐counterfeiting applications.

### 3.2 Main‐Chain Unzipping

Chain unzipping (Figure 3b) is a polymer degradation mechanism that is more controlled (i.e., directional, proceeding from one chain end to the other) and, as a result, less common than random chain scission. It has been exploited to develop smart materials such as self‐immolative polymers [90] and has potential for sequencing but also editing synthetic digital polymers [17]. For instance, sequence‐defined poly(urethanes) can undergo kinetically controlled depolymerization through a cyclization cascade, releasing 2‐oxazolidinone units that can be monitored for sequencing [91, 92, 93]. In this approach, chain unzipping is initiated by a terminal OH group and requires basic conditions and elevated temperatures to proceed. In a recent study [94], Anslyn and colleagues demonstrated a sophisticated approach where the unzipping of oligourethane sequences can be selectively triggered using protecting groups that function as chemical locks. They showcased this by mixing resins immobilizing various sequences and triggers, achieving and confirming the selective release of a specific sequence through sequencing. More recently, Zhang and coworkers introduced an interesting example of fully erasable, rewritable, and editable digital poly(γ‐butyrolactone)s [95]. These polymers are synthesized via a sequence‐defined polymerization strategy using γ‐butyrolactone and α‐methyl‐γ‐butyrolactone as a binary alphabet. The encoded information can be erased through optimized unzipping depolymerization, regenerating the parent monomers. These recovered monomers can be reused to synthesize new polymers, thereby enabling a rewriting capability.

### 3.3 Main‐Chain Substitution

In 2020, our group expanded the unzipping concept described above by introducing a damage/repair mechanism (Figure 3c) for editing sequence‐defined poly(*N*‐substituted urethane)s [96]. In this work, oligomers and polymers with defined lengths and monomer sequences were synthesized via a two‐step process: first, the formation of an activated carbonate, followed by a chemoselective reaction with the secondary amine group of an amino alcohol building block. SEC and ESI‐MS studies revealed that, under basic conditions at room temperature, OH‐terminated poly(*N*‐substituted urethane)s degrade via an ω→α unzipping mechanism. Interestingly, the degradation behavior depends on the composition of the polymer chain, allowing the content of an informational sequence to be efficiently tuned through monomer design. For instance, polymers with a hexyl carbamate terminal unit show no notable degradation under basic conditions. Nevertheless, if the hexyl carbamate unit is embedded within an ethyl carbamate sequence, partial depolymerization occurs from the ω‐terminus up to the hexyl unit, enabling selective erasure of specific information sections. This selective depolymerization property is particularly valuable for anti‐counterfeiting applications involving molecular barcodes which can be purposely altered under specific conditions, enhancing security. Furthermore, if the degraded chain retains reactive ends (α‐COOH, ω‐OH), the erased section can be reconstructed or replaced with a new sequence using simple modification conditions, unlocking unprecedented possibilities for molecular data editing, correction, and rewriting. Du Prez and coworkers have also reported an elegant strategy that involves reversibly modulating the position of a specific tag within sequence‐encoded oligo(thioether urethane)s. The efficiency of the method relies on the orthogonal cleavage of a thioester moiety by an amidation reaction, while a new label can be readily added via a thioesterification. This approach allows for the reversible insertion and deletion of data within an existing piece of information [97].

### 3.4 Side‐Chain Scission

Besides main‐chain mechanism, controlled side‐chain scission (Figure 3d) may also be exploited to edit the informational content of digital polymers. This was for example demonstrated by our group using light as a trigger [98]. In this work, sequence‐encoded poly(phosphodiester)s (PPDEs) containing light‐sensitive side‐chain moieties (*o*‐nitrobenzyl and *o*‐nitroveratryl), defined as 0‐bit and 1‐bit, respectively, were prepared by automated solid‐phase phosphoramidite chemistry [99]. It was observed that under light exposure, these photolabile groups are cleaved, converting the polymer into a non‐coded homopolymer, while preserving the integrity of the polymer backbone (photo‐erasing concept). A photo‐revealing concept was also examined with a series of digitally‐encoded PPDEs, containing both light‐sensitive and light‐inert side‐chain moieties (*o*‐ and *p*‐nitrobenzyl, respectively). Since these moieties share the same molar mass, the sequence remains undecodable by MS/MS spectrometry. Nevertheless, upon light exposure, the *o*‐nitrobenzyl side chains (0‐bit) are cleaved, while the *p*‐nitrobenzyl motifs (1‐bit) remain intact. This creates a mass differential, allowing the sequence to be decoded by MS/MS and revealing a hidden message. A novel strategy for bit‐level editing and site‐specific mutations in sequence‐encoded PPDEs was also investigated in this study, drawing inspiration from irradiation‐mediated mutagenesis. In this case, the binary encoding alphabet was expanded by introducing a 0‐bit monomer and two isobaric 1‐bit monomers (one containing a light‐sensitive moiety and the other a light‐inert moiety). MS/MS sequencing spectra obtained before and after light exposure confirmed that upon light irradiation the light‐sensitive 1‐bit could be selectively converted to a 0‐bit, while the light‐inert 1‐bit remained unchanged.

### 3.5 Full‐Chain Displacement

Beyond bit‐level chain editing (Figure 1c), synthetic digital polymers can also be edited at the file‐level. Very recently, our group demonstrated that precise file‐level editing can be achieved by designing TMSD reactions that target specific digital polymer segments within bio‐hybrid assemblies [100]. To achieve this, automated phosphoramidite synthesis was used as a reliable method for the preparation of uniform bio‐hybrid macromolecules composed of synthetic, sequence‐encoded PPDEs segments and ssDNA oligonucleotides [88]. A key advantage of this novel approach lies in the versatility of monomer selection, which enables the construction of synthetic polymer segments with high information density and tunable stability [101]. In the TMSD editing process [100], DNA is only used as a supramolecular connector and its informational sequence is not exploited for data storage. In other words, only the synthetic informational segments are edited. The DNA component acts as a multifunctional tool enabling both the structural organization and exchange of synthetic polymer strands, which can be monitored and confirmed via techniques like polyacrylamide gel electrophoresis (PAGE). Although DNA allows for highly precise control over which strands are displaced and when, strand displacement is highly sensitive to toehold length, ionic strength, temperature, and pH, therefore, careful optimization of these conditions is essential to ensure efficient and reliable operation. Additionally, while TMSD reactions are not inherently irreversible, their ability to be parallelized allows simultaneous targeting of multiple sites, facilitating complex, multistep reconfiguration within the system. For example, by introducing specific input strands it is possible to selectively erase or rewrite digital content within a bio‐hybrid assembly, through parallel or series TMSD reactions (PTMSD or STMSD) [102]. Beyond the demonstrated selective release and precise rewriting of information, this bio‐hybrid approach offers significant potential for more advanced operations, particularly within 2D and 3D architectures that enable substantially increased storage density. It can also be adapted so that the TMSD‐generated information content (output) can be isolated from a bulk solution, enabling subsequent sequencing and decoding. Still, although strand displacement is a powerful, non‐destructive strategy for file‐level editing, it is constrained by critical limitations. Particularly, thermodynamic trade‐offs between stability and reversibility make it increasingly difficult to maintain reliable performance as system complexity scales.

## 4 Conclusion

The field of macromolecular data storage is rapidly evolving and is no longer limited to passive storage, as underlined in this minireview. Chemists, biologists, computer scientists, and experts in nanotechnology are working together to design innovative methods and optimized strategies that enable dynamic data management at the bit, file, and library levels. Currently, DNA‐based data storage stands as the most flexible and reconfigurable macromolecular storage option. The maturity of biotechnological tools, such as PCR, epigenetic modifications, and gene editing, enables a wide range of active data storage capabilities. Similar to silicon‐based storage, DNA media can be dynamically reprogrammed, allowing for data rewriting, editing, and even computation. In contrast, synthetic digital polymers, that have been less explored, offer fewer options for managing and editing large datasets. However, as highlighted in the second part of this review, their greater chemical diversity may provide unique advantages for information processing at the primary structural level.

Despite these advancements in macromolecular data storage, significant challenges remain. Writing and reading speeds continue to be a major bottleneck, limiting the field's scalability for big data applications. Information retrieval also poses a critical hurdle, and while promising progress has been made, the compatibility of current sequencing methods with dynamic operations must be addressed in future research. This is particularly relevant for synthetic digital polymers, which still rely on early‐stage sequencing technologies. Nevertheless, the rapid evolution of this field is remarkable. Most of the examples discussed in this minireview have emerged within the last five years, and further breakthroughs are therefore anticipated in a near future.

## Author Contributions

**Jakub Ossowski**: writing – original draft, writing – review and editing. **Oskar Affenberger**: writing – original draft, writing – review and editing. **Jean‐François Lutz**: writing – original draft, writing – review and editing, supervision, funding acquisition. **Maria Nerantzaki**: writing – original draft, writing – review and editing, supervision.

## Conflicts of Interest

The authors declare no conflicts of interest.

## Data Availability

Data sharing not applicable to this article as no datasets were generated or analyzed during the current study.
